# The Thirty-Eighth Annual Commencement of the Baltimore College of Dental Surgery

**Published:** 1878-04

**Authors:** 


### EDITORIAL, ETC.

---

*The Thirty-Eighth Annual Commencement of the Baltimor College of Dental Surgery* was held on the evening of March 5th, 1878, at the Academy of Music, Baltimore City, before a large and brilliant audience, the auditorium being completely filled. The stage, as is usual on such occasions, was reserved for the Faculty, Graduating Class and Guests. The Music included fine operatic and popular overtures, &c., and was rendered by the Fifth Regiment Orchestra, Wernig, director.

The opening prayer was offered by the Rev. J. W. Kaye. Prof. F. J. S. Gorgas, Dean of the College, then read the Mandamus under which the institution was authorized to confer upon the graduates the degree of "Doctor of Dental Surgery," and also announced the names of those who had complied with the requirements of the Charter, and passed satisfactory examinations on the different branches necessary to qualify students for the practice of Dentistry. After music the ceremony of conferring the degrees on the members of the Graduating Class was proceeded with, the members advancing in classes and receiving their diplomas as testimonials at the hands of the Dean.

The display of beautiful floral favors for the graduates was unusually large and fine, and their presentation greatly enjoyed by the large audience present. The Valedictory Address was then delivered by Rev. Julius E. Grammer, D. D., who passed in review the progress in the science of dental surgery, the usefulness of the Baltimore College of Dental Surgery, having been organized as the first College of the kind in the world and as an experiment, but that it had a substantial basis in the necessities of the human race, and was in answer to the demand for progress in useful and beneficent sciences. That the modest institution which was founded in 1839, has become an influence throughout civilization, and has contributed to bring about a remarkable development in dental science. That this College can point with pride to the high standing of many of its graduates, and the prominent stations they command as the leading practitioners in this country and Europe. This address was listened to with the greatest attention, and the speaker warmly applauded at its close. The Class Address was then delivered by James Williams Tucker, of Virginia, a member of the Graduating Class, who for thirty-four minutes held the earnest attention of the large audience, and was many times applauded. The Benediction was then pronounced and the Thirty-Eighth Session of this Mother of Dental Colleges was thus appropriately closed.

The following Graduates had conferred upon them the Degree of Doctor of Dental Surgery, completing the number of 800 Alumni of this institution :

| | Subject of Thesis. |
|---|---|
| Jose Dolores Amieva, Jr., | *West Indies.*—Mercurial Stomatitis. |
| George Edward Baughman, | *Maryland.*—Digestion. |
| Friedrich Moritz Brandt, | *Germany.*—Leptothrix Buccalis. |
| James Thomas Calvert, | *South Carolina.*—The Dental Pulp. |
| Elvira Castner, | *Germany.*—Second Dentition. |
| Adolfine Petersen, | *Germany* —First Dentition. |
| William P. Crickenberger, | *Virginia.*—Respiration. |
| Charles Simon Hoffman, | *Maryland.*—Caries of the Teeth. |
| Charles Seymour Kelly, | *Montana Ter.*—Dental Caries. |
| Joseph Cicero King, | *Tennessee.*—Mastication. |
| John William Daniel Maier, | *Maryland.*—Diseases of the Gums. |
| Alexander Carson McCurdy, | *Pennsylvania.*—Dislocation of Inferior Maxillary. |
| Granville Eustace Medley, | *Kentucky.*—Circulation of the Blood. |
| William Henry Richards, | *Tennessee.*—Nitrous Oxide Gas. |
| James Williams Tucker, | *Virginia.*—Filling Teeth. |
| Robert Bentley Varden, | *Maryland.*—The Blood. |
| John Nathan Webster | *Virginia.*—The Ideal Dentist. |

The Thirty-Ninth Annual Session will commence on the 15th of October, 1878, and continue until March, 1879. The Summer Session will commence April 1st, and continue until the last of June, 1878. The Infirmary is open during the entire year for dental operations.

